# Lymphangiectasia and genital lymphedema secondary to metastatic Crohn’s^☆^

**DOI:** 10.1016/j.abd.2025.501150

**Published:** 2025-07-03

**Authors:** Nelly Marlene Román Mendoza, María Dolores Caro Gutiérrez, José Javier Mateos Rico, Alberto Alegre Bailo, Lourdes Estrada Muñoz, Francisco Javier Vicente Martín

**Affiliations:** a Department of Medical-Surgical Dermatology and Venereology, Hospital Universitario Rey Juan Carlos, Móstoles, Madrid, Spain; b Department of Pathological Anatomy, Hospital Universitario Rey Juan Carlos, Móstoles, Madrid, Spain

*Dear Editor,*

A 34-year-old male with Crohn’s disease (CD) diagnosed in 2006 and treated with adalimumab since 2022, presented with persistent genital lymphedema (GL) starting in 2013. Extensive serological testing for sexually transmitted infections (STIs), autoimmune markers, and imaging studies were performed, leading to a diagnosis of idiopathic GL in 2018. The patient underwent circumcision in 2019, but the procedure did not improve his symptoms. In 2023, he was referred to dermatology due to genital warts of one year’s duration.

On physical examination, genital swelling, predominantly involving the penis, was observed, causing distal deformity often described as “saxophone penis” (Fig. 1A). Multiple skin-colored popular lesions, 3‒5 mm in size, were identified on the penile shaft and scrotum, some of which were umbilicated, while others were pedunculated (Fig. 1B). Histological examination of one papule revealed dilated vascular channels in the papillary dermis. Immunohistochemical staining demonstrated positive expression of D2-40 (podoplanin) and CD31 by the endothelial cells, confirming the lymphatic origin of the lesions (Fig. 2). Based on these findings, the lesions were diagnosed as lymphangiectasia secondary to GL. Further serological tests for STIs, autoimmunity, and blood parasites returned negative results. Imaging with ultrasound and abdominopelvic CT revealed edema of the penile subcutaneous tissue (SCT). A biopsy of the penile base demonstrated lymphoplasmacytic perivascular infiltrates in the deep dermis and SCT, with no evidence of microorganisms (Fig. 3). Considering the history of CD, the subsequent development of GL with lymphangiectasia, and the histological findings, the condition was classified as GL secondary to CD or anogenital CD, a form of metastatic Crohn's disease (MCD).

**Figure 1:**
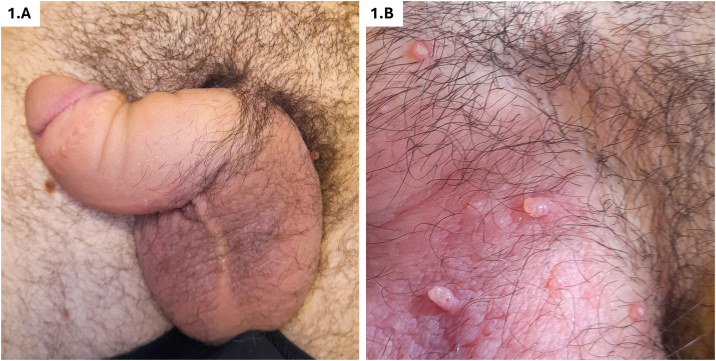
(A) Genital edema predominantly involving the penis. Distal deformity often referred to as “saxophone penis”. (B) Skin-colored popular lesions located on the penile shaft and scrotum.

**Figure 2:**
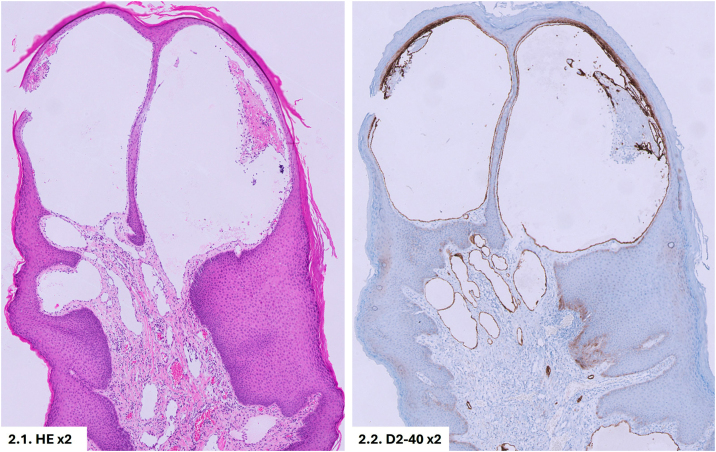
(1) Hematoxylin-eosin staining of a papular lesion, showing dilated vascular channels in the papillary dermis (Hematoxylin & eosin, ×2). (2) Immunohistochemical staining with D2-40 positive in endothelial cells, confirming the lymphatic origin of the vascular structures (D2-40 ×2).

**Figure 3:**
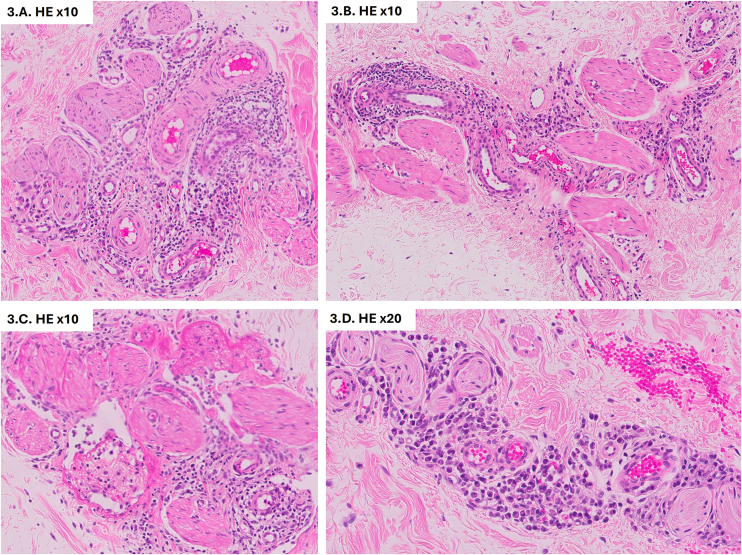
(A‒C) Lymphoplasmacytic perivascular infiltrates observed in the deep dermis and subcutaneous tissue of the penile base (Hematoxylin & eosin, ×10). (D) Detailed view of lymphoplasmacytic perivascular infiltrates (Hematoxylin & eosin, ×20).

Lymphangiectasias are dilated lymphatic vessels in the superficial dermis, typically occurring in areas of pre-existing lymphedema. These vessels are confirmed by positive immunohistochemical staining for podoplanin (D2-40), a specific marker for lymphatic endothelial cells. In the literature, the terms lymphangiectasia and lymphangioma are often confused and used interchangeably, which can lead to diagnostic confusion.^1^, ^2^ Penile lymphedema is a rare, chronic condition that may be idiopathic or secondary to causes such as neoplasms, surgeries, radiation, infections (e.g., STIs, filariasis, tuberculosis), sarcoidosis, hidradenitis suppurativa or CD.^3^ Diagnostic tools include ultrasound, CT or MRI of the abdomen and pelvis, lymphoscintigraphy, and screening for STIs, filariasis, and angiotensin-converting enzyme levels. Biopsy is recommended when specific etiologies are suspected.^3^, ^4^, ^5^

Anogenital Crohn's disease (AGCD), also referred to as metastatic Crohn's disease or anogenital granulomatosis (AG), is a rare condition that is challenging to diagnose, particularly in the absence of gastrointestinal symptoms.^6^, ^7^, ^8^ MCD involves granulomatous inflammation in skin areas non-contiguous to the gastrointestinal tract and often presents as nodules, plaques, or ulcerative lesions, primarily affecting the extremities, genitalia, or perianal region.^7^, ^9^ In our case, this rare presentation highlights the diagnostic challenges associated with AGCD. A systematic review of AGCD found that genital edema was the most common clinical manifestation in both males and females.^6^ Histological findings may include granulomas (seen in up to 70% of cases), lymphocytic and plasma cell inflammatory infiltrates, and occasionally lymphangiectasia.^6^ In this case, the absence of granulomas does not rule out the diagnosis of MCD if other characteristic features are present, and the use of anti-TNF therapy (adalimumab) could have suppressed granuloma formation.

A study by Shim et al. described 41 cases of GL, including 10 patients with a prior diagnosis of CD, and four diagnosed with CD during the evaluation of their lymphedema. Most of these patients had no gastrointestinal symptoms, and GL was considered a manifestation of MCD. GL typically appears years after CD diagnosis in adults but may be the first manifestation in pediatric patients.^6^, ^10^

Treatment of AGCD is challenging due to the absence of standardized guidelines. Management options include oral antibiotics, biologics (anti-TNF, ustekinumab), and JAK inhibitors.^6^, ^10^ Surgical interventions, such as lymphatic drainage or lesion excision, can improve symptoms but do not address the underlying cause.^6^

In conclusion, AGCD is a rare condition with frequent diagnostic delays. Genital edema is its most frequent presentation and, in some cases, the first manifestation of CD. Early recognition of GL and lymphangiectasia in patients with CD is crucial to avoid unnecessary surgical interventions. Multidisciplinary management involving imaging, histopathology, and clinical expertise is essential for optimal outcomes.

## Financial support

None declared.

## Authors' contributions

Nelly Marlene Román Mendoza: Writing of the manuscript or critical review of important intellectual content; critical review of the literature.

María Dolores Caro Gutiérrez: Writing of the manuscript or critical review of important intellectual content; critical review of the literature; final approval of the final version of the manuscript.

José Javier Mateos Rico: Final approval of the final version of the manuscript.

Alberto Alegre Bailo: Critical review of the literature.

Lourdes Estrada Muñoz: Critical review of the literature; final approval of the final version of the manuscript.

Francisco Javier Vicente Martín: Final approval of the final version of the manuscript.

## Conflicts of interest

None declared.
